# Effect of Germinated Pigmented Rice “Superjami” on the Glucose Level, Antioxidant Defense System, and Bone Metabolism in Menopausal Rat Model

**DOI:** 10.3390/nu11092184

**Published:** 2019-09-11

**Authors:** Soo Im Chung, Tae-ho Ham, Mi Young Kang

**Affiliations:** 1 International Agricultural Training Center, Kyungpook National University, Daegu 41566, Korea; zizibe0312@nate.com; 2 Department of Food Science and Nutrition, Brain Korea 21 Plus, Kyungpook National University Daegu 41566, Korea; 3 Institute of Life and Environment, Konkuk University, Seoul 05029, Korea; lion78@daum.net

**Keywords:** Superjami, menopause, antioxidant, hyperglycemia, bone turnover

## Abstract

Women experience physical, mental, and social changes during menopause. It is important to maintain a healthy diet for effective menopause management. The effect of germinated Superjami, a deep violet colored rice cultivar, on the body weight, glucose level, antioxidant defense system, and bone metabolism in a menopausal rat model was investigated. The animals were randomly divided into three groups and fed with a normal diet (ND), a control diet supplemented with 20% (*w*/*w*) non-germinated Superjami flour (NGSF), or germinated Superjami flour (GSF) for eight weeks. The NGSF and GSF groups exhibited significantly lower body weight and fat, glucose and insulin contents, adipokine concentrations, and bone resorption biomarker levels, and higher antioxidant enzyme activities and 17-β-estradiol content than the ND group (*p* < 0.05). The GSF group showed greater glucose homeostasis, antioxidative, and bone metabolism-improving effects compared with the NGSF group. These findings demonstrate that germination could further improve the health-promoting properties of Superjami and that this germinated pigmented rice cultivar could be useful in the treatment and management of menopause-induced hyperglycemia, oxidative stress, and bone turnover imbalance.

## 1. Introduction

Superjami, a newly developed pigmented rice cultivar with a deep violet pericarp, has been recently shown to have antioxidative, glucose homeostatic, hypolipidemic, and body weight-lowering effects [1]. Superjami contains high amounts of anthocyanins, such as cyanidin-3-glucoside, which are known for their strong antioxidant properties [2]. Pigmented rice cultivars, particularly those with a dark-colored pericarp like Superjami, are rich in phytochemicals, including phenolic and flavonoid compounds, and have high reducing power and free radical scavenging ability [3,4]. The dietary consumption of pigmented rice has been associated with improved lipid and glucose profiles, and a reduced risk of diabetes and cardiovascular disease in both humans and laboratory animals [5,6].

Studies indicate that the nutritional value and health-promoting properties of brown rice can be enhanced by germination [7,8]. Germinated rice contains larger quantities of bioactive compounds, including tocopherols, γ-oryzanol, phenolic compounds, and γ-aminobutyric acid (GABA), than non-germinated rice [9,10]. Other cereal grains, like oat, wheat, and barley, also exhibit a significant increase in nutrients and bioactive compounds after germination [11,12]. The process of germination involves soaking brown rice in water for a few days, which causes the softening of the endosperm and breaking down of the cell walls surrounding various compounds, leading to the release of free and bound materials, and increased nutrient bioavailability [13]. Moreover, dormancy enzymes are activated during rice germination, resulting in the degradation of large biomolecules and the production of bioactive substances [14]. Germination has been found to increase the quantity of antioxidant compounds and enhance the antioxidant capacity of Superjami rice [15]. Further, dietary supplementation of germinated Superjami rice has been previously shown to ameliorate the lipid metabolism and reduce the risk of dyslipidemia in menopause animal model [16].

Menopause, which is the permanent cessation of menstruation in women, has been associated with an increased risk of metabolic disorders and cardiovascular disease [17]. Past studies have revealed that menopause could cause an increase in the levels of cholesterol, triglyceride, glucose, and insulin in postmenopausal women due to a lack of ovarian hormones, particularly estrogen [17,18]. The rapid decrease in estrogen during menopause has also been linked with oxidative stress and advanced osteoporosis, a chronic bone disease characterized by an increased risk of fracture and loss of bone mineral density [19]. Ovariectomy or removal of the ovaries in experimental animals imitates estrogen deficiency in women after menopause. Recent studies have demonstrated that germinated Superjami rice could decrease the lipid levels in ovariectomized rats [16]. The current study was conducted to further research the potential curative effects of this germinated rice cultivar against metabolic dysfunction induced by menopause. The study aims to investigate the effect of germinated and non-germinated Superjami rice on the glucose level, antioxidant defense system, and bone metabolism in a postmenopausal rat model. 

## 2. Materials and Methods

### 2.1. Rice Sample Preparations

Unpolished rice grains of Superjami, provided by the Department of Agricultural Science, Korea National Open University, were grown in Gimje, Jeollabukdo, Korea. To remove dirt that may interfere with germination, the grains were washed lightly with distilled water three times. Then, 100 g of grains was evenly distributed in stainless-steel containers (350 mm × 325 mm × 60 mm; width × length × height) laid with cotton pads sufficiently soaked with distilled water, and subsequently incubated at 28 °C for three days to allow germination. During this time, the grains were checked twice a day for the absence of fungal infection or odor [20]. The germinated grains were dried at 45 °C for 3 h, to make fine flour using a grinding machine (SMX-750BH, Shinil Industrial Co., Ltd., Chungcheongnamdo, Korea), and stored at −20 °C until further analysis. The proximate composition of each rice flour was analyzed by adopting the Association of Official Analytical Chemists (AOAC) methods for measuring the contents of crude protein (Kjeldahl method), fat (Soxhlet method), ash (incineration at 525 °C), and moisture (oven-drying at 105 °C) [21].

### 2.2. The measurement of Bioactive Compounds

The measurements of γ-Oryzanol were taken from 6 mL of methanol with 1 g of rice sample, using a high-performance liquid chromatography (HPLC) system (1200 Series, Agilent Technologies, Waldbronn, Germany) equipped with a C18 column. The detection wavelength was set at 330 nm. The mobile phase consisted of methanol, acetonitrile, dichloromethane, and acetic acid (50:44:3:3, *v*/*v*/*v*/*v*), and the flow rate was 1.4 mL /min [22,23]. GABA contents were extracted using 70% ethanol at 10 times the volume of the sample and were then measured using an amino acid analyzer (L-8900, Hitachi High Technologies America, Inc., Schaumburg, IL, USA) [24]. Quercetin and ferulic acid were measured using an Agilent 1200 Series HPLC apparatus, with methanol and 5% acetic acid in water (*v*/*v*) as the mobile phase at a flow rate of 1.0 mL/min. The column temperature was 40 °C, and the detection wavelength was set at 380 nm [25]. The results are presented in Table 1. All chemicals in this study were HPLC grade or analytical grade (Merck, Darmstadt, Germany; Sigma–Aldrich, Inc., Steinheim, Germany).

### 2.3. Animals and Diets

Three-month-old, ovariectomized (OVX) Sprague Dawley rats (n = 30), purchased from Central Lab (Seoul, Korea) and weighing approximately 230 g each, were individually housed at 25 ± 2 °C and 50% relative humidity under a 12/12-h light–dark cycle. The rats were fed a normal chow diet for a week of acclimation and were then randomly divided into three dietary groups (n = 10): normal control diet (ND), and ND diet supplemented with either 20% (*w*/*w*) non-germinated Superjami flour (NGSF) or germinated Superjami flour (GSF). The composition of the experimental diet in each group is shown in Table 2 and was based on the AIN-93M diet [26]. The animals were allowed free access to food and water for eight weeks. At the ninth week of the experiment, experimental animals were given inhalation anesthesia by CO_2_ after 12 h of fasting. Blood samples were collected in tubes coated with heparin and centrifuged at 1200× *g*, 4 °C for 10 min in order to obtain the plasma.

The liver, kidney, and white adipose tissues were removed, rinsed with physiological saline, weighed, frozen in liquid nitrogen immediately, and stored at −70 °C until analysis. This study protocol was approved by the Ethics Committee of Kyungpook National University Industry Foundation (approval number 2016-0117).

### 2.4. Determination of Glucose, Insulin, and Adipokine Concentrations

The fasting glucose was determined using Accu-Chek Active Blood Glucose Test Strips (Roche Diagnostics, Berlin, Germany), and the plasma insulin was measured using a Rat Insulin ELISA kit (MyBioSource, San Diego, CA, USA). Commercial ELISA kits were purchased for assaying the plasma adipokines: resistin (MyBioSource), leptin (MyBioSource), adiponectin (MyBioSource), and tumor necrosis factor-α (TNF-α; Fine Test, Wuhan, China).

### 2.5. Measurement of Antioxidant Enzyme Activities

The hepatic and nephritic enzyme sources were prepared by homogenizing the liver or kidney (0.3 g) in a buffer solution containing 0.1 M triethanolamine, 0.02 M, and 2 mM dithiothreitol (DTT) [27]. The liver or kidney mixture was then centrifuged at 1000× *g*, 4 °C for 15 min, and the supernatant was further centrifuged at 10,000× *g*, 4 °C for 15 min to remove the mitochondrial fraction. The supernatant was then ultra-centrifuged at 103,000× *g*, 4 °C for 1 h to obtain the cytosol supernatant fraction, which was used for superoxide dismutase (SOD), glutathione peroxidase (GPx), and glutathione reductase (GR) determinations, and the pellet of microsome, which was used for analyzing the paraoxonase (PON) activity. The pellet was combined with buffer solution and centrifuged at 12,000× *g*, 4 °C for 20 min to obtain the mitochondrial fraction, which was used for catalase (CAT) assessment. The protein content was measured by the Bradford assay [28]. Meanwhile, to measure erythrocyte enzyme activity, the plasma was removed, and the buffy coat cells were washed three times with physiological saline.

The antioxidnat enzyme activities were measured by spectrophotometric assays. SOD activity was determined by the inhibition of pyrogallol autoxidation at 420 nm. The mixture (50 mM Tris–HCl buffer pH 8.5, 7.3 mM pyrogallol) with cytosol or erythrocyte (90 μL) was reactivated at 25 °C for 10 min, and then the change in absorbance was measured [29]. For evaluating GPx activity, the assay mixture (0.05 M Tris–HCl buffer pH 7.2, with 20 mM glutathione, 5 mM NADPH, and 10 mM H_2_O_2_) was incubated at 25 °C for 5 min, and the decreased absorbance of NADPH was measured when the glutathione disulfide was reduced by GR and NADPH at 340 nm [30]. CAT activity was determined by recording the decomposition of H_2_O_2_ in the reaction mixture (0.05 M potassium phosphate buffer pH 7.4, with the mitochondrial or erythrocyte fraction) at 240 nm for 5 min [31]. GR activity was analyzed using potassium phosphate buffer (pH 7.4) containing 1 mM EDTA, 1 mM glutathione disulfide, 0.1 mM NADPH, and the cytosol or erythrocyte fraction. Enzyme activity units were expressed as nmol or µmol oxidized NADPH/min/mg protein or hemoglobin [32]. PON activity was quantified by measuring the rate of formation of *p*-nitrophenol via decomposition of paraoxon through the reaction of 100 mM Tris–HCl buffer (pH 8.0) with the microsome fraction, 2 mM CaCl_2_, and 15 mM paraoxon [33].

### 2.6. Analysis of Biochemical Markers of Bone Metabolism

Plasma 17-β-estradiol was measured using an ELISA test kit (ab108667, Abcam, Cambridge, UK). Intact parathyroid hormone (PTH; MBS031256), osteocalcin (MBS 728975), N-terminal telopeptide of type 1 collagen (NTx-1; MBS 727573), and C-terminal telopeptide of type 1 collagen (CTx-1; MBS 9901667) were assayed using commercial ELISA test kits supplied by MyBioSource. Calcium was measured using a calcium detection assay kit (ab102505) supplied by A bcam.

### 2.7. Statistical Analysis

All data are presented as the mean ± standard error (SE). The data of bioactive compounds were analyzed by paired t-test, and animal studies were evaluated by one-way analysis of variance, followed by Tukey’s test using the Statistical Package for Social Sciences software program version 21.0 (SPSS, Inc., Chicago, IL, USA). Statistical significance was set at *p* < 0.05.

## 3. Results

### 3.1. Bioactive Compounds

Germinated Superjami showed a significant increase in γ-oryzanol compared with Superjami, and a more than two-fold increase in GABA contents. In addition, germinated Superjami exhibited significantly higher amounts of quercetin and ferulic acid than Superjami.

### 3.2. Body Weight Gain

The ND group had a significantly higher body weight gain (177 g) and white adipose tissue weight (3.01 g/100 g body weight) than the rice-fed groups (Table 3). The GSF rats exhibited the lowest body weight gain (127 g) and body fat weight (2.37 g). There was no significant difference in the feed intake and energy intake among the animal groups.

### 3.3. Glucose, Insulin, and Adipokine Levels

The initial blood glucose level was not significantly different among the animal groups (Table 4). At the end of the experimental period, the GSF group exhibited markedly lower levels of blood glucose (101 mg/dL) and plasma insulin (9.14 ng/L) than the other groups. The ND rats showed the highest glucose (118 mg/dL) and insulin (14.0 ng/L) levels. The amount of plasma adiponectin was significantly higher in the GSF group (1.09 ng/mL) than the NGSF (0.68 ng/mL) and ND (0.25 ng/mL) groups. On the contrary, the leptin, resistin, and TNF-α contents were lowest in the GSF group (4.14 ng/mL, 15.2 ng/mL, and 4.48 µg/mL, respectively) and highest in the ND group (4.92 ng/mL, 31.2 ng/mL, and 9.99 µg/mL, respectively).

### 3.4. Antioxidant Enzyme Activities

The activities of hepatic, nephritic, and erythrocyte enzymes SOD, GPx, CAT, GR, and PON were highest in the GSF-fed animals, followed by the NGSF group, and then the NC group (Table 5). Among the antioxidant enzymes analyzed, GR had the highest activity in the rice-fed animals, particularly the GSF group.

### 3.5. Bone Metabolism Biochemical Markers

**Figure 1:**
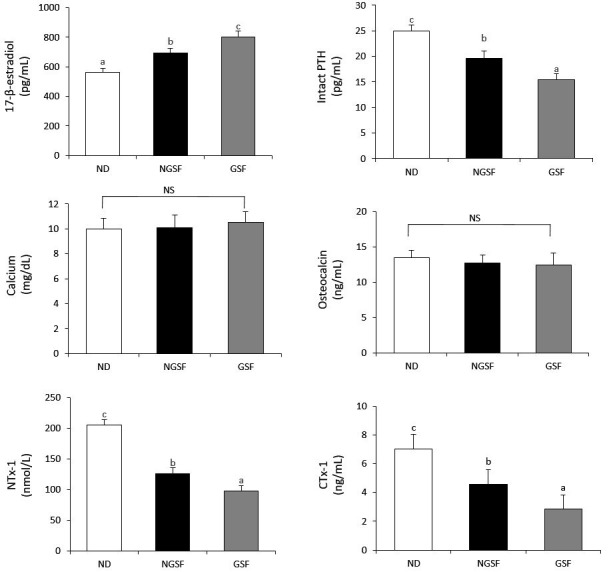
Biomarkers of bone metabolism in ovariectomized rats fed with non-germinated Superjami rice flour or germinated Superjami rice flour. Error bars correspond to the standard error of the mean. Means not sharing a common superscript are significantly different at *p* < 0.05. ND, normal diet (AIN-93M); NGSF, ND + non-germinated Superjami flour; GSF, ND + germinated Superjami flour; PTH, parathyroid hormone; NTx-1, N-terminal telopeptide of type 1 collagen; CTx-1, C-terminal telopeptide of type 1 collagen. NS, not significantly different; ^a–c^ Means not sharing a common superscript are significantly different among the group at *p* < 0.05.

The 17-β-estradiol level was highest in the GSF group (803 pg/mL) and lowest in the ND group (562 pg/mL) (Figure 1). On the contrary, the levels of intact PTH, NTx-1, and CTx-1 were lowest in the GSF group (15.5 pg/mL, 98.2 nmol/L, and 2.85 ng/mL, respectively) and highest in the NC group (25.0 pg/mL, 206 nmol/L, and 7.04 ng/mL, respectively). The amounts of calcium and osteocalcin were not significantly different among the groups.

## 4. Discussion

The absence of estrogen in postmenopausal women is known to promote metabolic disorders and increase the risk of dyslipidemia, diabetes, obesity, and osteoporosis [17,19]. OVX rats, which mimic the estrogen-deficient condition in women, have been widely used in examining the physiological changes caused by menopause and the potential treatment approaches that could prevent menopause-induced metabolic dysfunctions. In the current study, the effects of dietary feeding of germinated Superjami, a new deep-violet rice cultivar, on the body weight, glucose and adipokine levels, antioxidant enzyme activities, and bone metabolism in OVX rats were determined. Results showed that both the germinated and non-germinated Superjami rice flour significantly decreased the body weight gain, body fat, glucose and insulin levels, and adipokine concentrations and markedly improved the antioxidant defense system and bone metabolism in OVX rats. A previous study also revealed that Superjami rice has strong glucose homeostasis and antioxidative effects in high fat-fed mice [1]. Ethanolic extracts from Superjami rice bran have reduced the body weight and improved the glucose and lipid metabolisms in OVX rats as well [34]. Likewise, other pigmented rice varieties have decreased the body weight and glucose level and enhanced the antioxidant status in laboratory animals and human subjects [5,6].

The present study revealed that the germinated Superjami has greater body weight-lowering effect, glucose homeostasis and antioxidative activities, and bone metabolism-improving properties than the non-germinated sample. Germinated cereal grains have substantially higher nutrient content and bioactive compounds than their non-germinated counterparts [9,10,11]. This trend can be attributed to the release of free and bound materials resulting from the degradation of cell walls and activation of dormant enzymes involved in the synthesis of bioactive compounds during germination [14]. Germination significantly increased the amounts of γ-oryzanol, GABA, and phenolic compounds, such as quercetin and ferulic acid, in Superjami rice. The GABA content, in particular, increased by 25-fold after germination. These observations are consistent with a substantial increase in GABA, quercetin, and γ-oryzanol contents in pigmented rice after germination [10,24]. GABA, which is the component generated the most during germination, possesses various physiological effects, including anti-obesity, antidiabetic, and hypolipidemic [9,35]. γ-Oryzanol, tocopherol, tocotrienol, and phenolic compounds, such as ferulic acid, are antioxidant compounds that also have anti-obesity and anti-diabetic properties [24,36,37]. It could be that the improved glucose homeostasis, antioxidative, and body weight-lowering effects observed in the GSF group relative to NGSF rats occurred because of the increased amounts of bioactive compounds in germinated Superjami rice.

The reduction in blood glucose and insulin levels observed in rice-fed rats, particularly the GSF group, might have been associated with mechanisms involving the elevation of adiponectin concentration and decrease in leptin, resistin, and TNF-α levels in these animals. Adiponectin induces insulin-sensitizing effects, and its increased concentration could improve insulin sensitivity and glucose tolerance [38]. Moreover, an earlier study indicated that an enhanced level of adiponectin could protect women from developing diabetes after menopause [39]. The adipokines leptin, resistin, and TNF-α are also involved in glucose metabolism, but unlike adiponectin, their elevated levels are associated with the development of diabetes and obesity [38,40].

Antioxidant enzymes, such as SOD, GPx, CAT, GR, and PON, function as free radical-quenchers and they are part of a highly complex antioxidant defense system that is responsible for the regulation of oxidative stress [41]. In the present study, the activities of these antioxidant enzymes were substantially higher in Superjami rice-fed rats, especially the GSF group, compared with that of the control group, indicating a significant improvement in the antioxidant defense system, reducing the risk of ovariectomy-induced oxidative damage in rats fed with Superjami. SOD enzymatically transforms superoxide radicals into H_2_O_2_, which is then converted by GPx and CAT enzymes into non-toxic products [42]. GR enzyme transforms oxidized glutathione into reduced glutathione, an antioxidant [43], while PON enzyme reduces lipid peroxides and hydrolyzes oxidized phospholipids [44]. Superjami rice is rich in natural antioxidants, and germination further increases the amount of these antioxidant compounds, which may have been responsible for the enhanced antioxidant defense status in OVX rats [15]. Studies have shown that Superjami rice inhibited oxidative stress and enhanced the antioxidant enzyme activities in high fat-fed mice and that its antioxidant capacity markedly increased after germination [1,15].

The lack of estrogen in postmenopausal women could cause an imbalance between bone formation and resorption, which then results in a rapid rate of bone loss and increased bone fragility [19,45]. The relatively high amount of 17-β-estradiol, the most potent form of estrogen, found in the GSF group indicates that the germinated Superjami could suppress the ovariectomy-induced reduction of estrogen in OVX rats. The hormone 17-β-estradiol has been shown to decrease the rate of bone turnover and inhibit bone loss in postmenopausal women [46,47]. The levels of intact PTH, NTx-1, and CTX-1, which are biochemical markers of bone resorption, significantly decreased in the NGSF and GSF groups relative to the control group, suggesting reduced bone turnover and an improved bone metabolism in these animals. Oxidative stress plays a major role in the pathogenesis of osteoporosis [48,49]. Reactive oxygen specs (ROS) not only affect the maintenance and change in osteoblasts, osteoclasts, and bone mineral density, but also have a major contributory role in reducing bone resorption and bone mass, so increased antioxidant enzymes can alleviate osteoporosis in menopausal women [50]. Notably, the decrease in estrogen after menopause is highly correlated with ROS-induced osteoporosis [51].

Dietary antioxidants have been found to prevent bone loss in both OVX animal models and postmenopausal women and have been suggested as a potential treatment against osteoporosis [52,53,54]. Hence, the strong antioxidative property of Superjami rice, particularly the germinated grain, may have been partly responsible for the improved bone metabolism observed in the NGSF and GSF groups.

## 5. Conclusions

The dietary feeding of non-germinated and germinated Superjami rice powder markedly reduced the body weight, glucose and insulin concentrations, adipokine contents, and bone resorption biomarker levels and increased the antioxidant enzyme activities and 17-β-estradiol content in OVX rats. Germination for three days substantially increased the quantity of bioactive compounds, such as GABA, γ-oryzanol, quercetin, and ferulic acid, in Superjami, which could have been responsible for the greater glucose homeostasis, antioxidative, and bone metabolism-improving effects observed in the GSF group compared to those of the NGSF rats. Germinated Superjami may have potential beneficial effects in the treatment and control of postmenopausal hyperglycemia, oxidative damage, and increased bone turnover.

## Figures and Tables

**Table 1:** Proximate compositions and bioactives of Superjami.

Contents	Superjami	Germinated Superjami
Proximate composition (% dry basis)		
Protein	7.21 ± 0.55	6.75 ± 0.87
Fat	2.29 ± 0.14	3.34 ± 0.68
Ash	1.22 ± 0.08	1.19 ± 0.09
Moisture	12.49 ± 1.25 *	28.96 ± 3.22
Carbohydrate	76.79 ± 3.48 *	59.76 ± 4.09
Bioactives (μg/g rice)		
γ-Oryzanol	372.32 ± 98.21	670.42 ± 59.98 *
GABA	427.55 ± 35.58	10814.44 ± 117.55 *
Quercetin	14.52 ± 1.33	22.45 ± 2.21 *
Ferulic acid	70.43 ± 6.99	143.57 ± 15.95 *

Data are mean ± SE. * *p* < 0.05 Superjami vs. germinated Superjami by paired *t*-test.

**Table 2:** Diet compositions of animal experimental.

Ingredient (g)	ND	NGSF	GSF
Casein	140	123.52	121
Sucrose	100	100	100
Dextrose	155	155	155
Corn starch	465.69	287.42	294.10
Cellulose	50	50	50
Soybean oil	40	34.76	30.6
Mineral mix	35	35	35
Vitamin mix	10	10	10
L-Cysteine	1.80	1.80	1.80
Choline bitartrate	2.50	2.50	2.50
NGSF		200	
GSF			200
Total	1000	1000	1000
Total energy (kcal)	3.8	3.8	3.8

ND, normal diet (AIN-93M); NGSF, ND + non-germinated Superjami flour; GSF, ND + germinated Superjami flour.

**Table 3:** Body weight gain and adipose tissue weight in ovariectomized rats fed with germinated Superjami rice flour.

Variable	ND (*n* = 10)	NGSF (*n* = 10)	GSF (*n* = 10)
Initial body weight (g)	231.02 ± 1.65	232.48 ± 1.35	231.98 ± 1.75
Final body weight (g)	406.22 ± 4.53 ^c^	384.65 ± 5.06 ^b^	356.25 ± 4.32 ^a^
Body weight gain (g)	177.27 ± 6.06 ^c^	155.47 ± 3.62 ^b^	127.55 ± 4.09 ^a^
White adipose tissue weight (g/100 g body weight)	3.01 ± 0.13 ^b^	2.57± 0.11 ^a^	2.37 ± 0.18 ^a^
Feed intake (g/day)	22.88 ± 1.08	23.45 ± 0.98	22.24 ± 1.21
Energy intake (kcal/day)	87.3 ± 2.11	89.1± 1.98	86.4± 2.09
FER	0.04 ± 0.00 ^c^	0.03 ± 0.00 ^b^	0.02 ± 0.00 ^a^

Data are mean ± SE. ^a–c^ Means in the same row not sharing a common superscript are significantly different at *p* < 0.05. ND, normal diet (AIN-93M); NGSF, ND + non-germinated Superjami flour; GSF, ND + germinated Superjami flour; FER, body weight gain/energy intakes per day.

**Table 4:** Glucose profile and adipokine content in ovariectomized rats fed with non-germinated Superjami rice flour or germinated Superjami rice flour.

Variable	ND (*n* = 10)	NGSF (*n* = 10)	GSF (*n* = 10)
Glucose (mg/dL)	118.54 ± 0.05 ^c^	106.54 ± 1.03 ^b^	101.25 ± 1.43 ^a^
Insulin (ng/L)	13.98 ± 0.42 ^c^	11.22 ± 0.34 ^b^	9.14 ± 0.12 ^a^
Resistin (ng/mL)	31.25 ± 0.31 ^c^	25.96 ± 1.86 ^b^	15.25 ± 1.14 ^a^
Leptin (ng/mL)	4.92 ± 0.24 ^b^	4.58 ± 0.22 ^a^	4.14 ± 0.11 ^a^
Adiponectin (ng/mL)	0.25 ± 0.02 ^a^	0.68 ± 0.04 ^b^	1.09 ± 0.08 ^c^
Tumor necrosis factor-α (µg/mL)	9.99 ± 0.58 ^c^	7.09 ± 0.45 ^b^	4.48 ± 0.32 ^a^

Data are mean ± SE. ^a–c^ Means in the same row not sharing a common superscript are significantly different at *p* < 0.05. ND, normal diet (AIN-93M); NGSF, ND + non-germinated Superjami flour; GSF, ND + germinated Superjami flour.

**Table 5:** Activities of antioxidant enzymes in ovariectomized rats fed with non-germinated Superjami rice flour or germinated Superjami rice flour.

Variable	ND (*n* = 10)	NGSF (*n* = 10)	GSF (*n* = 10)
Superoxide dismutase	1.37 ± 0.09 ^a^	2.25 ± 0.02 ^b^	4.18 ± 0.03 ^c^
Glutathione peroxidase	1.09 ± 0.01 ^a^	2.08 ± 0.01 ^b^	3.48 ± 0.09 ^c^
Catalase	0.17 ± 0.00 ^a^	0.27 ± 0.02 ^b^	0.49 ± 0.01 ^c^
Glutathione reductase	2.01 ± 0.53 ^a^	10.78 ± 2.13 ^b^	18.59 ± 3.11 ^c^
Paraoxonase	0.12 ± 0.01 ^a^	0.26 ± 0.01 ^b^	0.48 ± 0.00 ^c^
Nephritic enzymes (nmol/min/mg protein)			
Superoxide dismutase	1.25 ± 0.05 ^a^	1.48 ± 0.01 ^b^	1.51 ± 0.02 ^b^
Glutathione peroxidase	0.21 ± 0.02 ^a^	0.64 ± 0.03 ^b^	2.11 ± 0.08 ^c^
Catalase	0.18 ± 0.01 ^a^	0.20 ± 0.00 ^b^	0.26 ± 0.00 ^c^
Glutathione reductase	0.89 ± 0.01 ^a^	2.53 ± 0.11 ^b^	4.57 ± 0.09 ^c^
Erythrocyte enzymes (µmol/min/mg hemoglobin)			
Superoxide dismutase	1.94 ± 0.00 ^a^	2.69 ± 0.11 ^b^	2.87 ± 0.09 ^b^
Glutathione peroxidase	0.17 ± 0.01 ^a^	0.48 ± 0.02 ^b^	1.06 ± 0.04 ^c^
Catalase	0.16 ± 0.02 ^a^	0.38 ± 0.02 ^b^	0.59 ± 0.01 ^c^
Glutathione reductase	1.83 ± 0.01 ^a^	8.59 ± 0.21 ^b^	11.65 ± 0.69 ^c^

Data are mean ± SE. ^a–c^ Means in the same row not sharing a common superscript are significantly different at *p* < 0.05. ND, normal diet (AIN-93M); NGSF, ND + non-germinated Superjami flour; GSF, ND + germinated Superjami flour.
